# The effects of mindfulness-based interventions on alleviating academic burnout in medical students: a systematic review and meta-analysis

**DOI:** 10.1186/s12889-024-18938-4

**Published:** 2024-05-27

**Authors:** Zhizhuo Wang, Peiyun Wu, Yutong Hou, Jing Guo, Cheng Lin

**Affiliations:** 1 Department of Rehabilitation Medicine, School of Health, Fujian Medical University, No. 1 Xuefu North Road, University town, Fuzhou, Fujian 350122 China; 2 Department of Pediatrics, School of Pediatrics, Kunming Medical University, No. 1168 Chunrong West Road, Yuhua Street, Kunming, Yunnan 650504 China

**Keywords:** Mindfulness, Burnout, Medical students, Emotional exhaustion, Cynicism, Academic inefficacy, Meta-analysis

## Abstract

**Background:**

Mindfulness-based interventions have been tested to be the effective approach for preventing/reducing burnout in medical students. Therefore, this systematic review and meta-analysis aimed to synthesize the scientific evidence and quantify the pooled effect of MBIs on the burnout syndrome in medical students.

**Methods:**

A comprehensive literature search was conducted in the databases, including PubMed, Embase, ERIC, PsycINFO, Scopus, Cochrane Central Register of Controlled Trials (CENTRAL), China National knowledge Information Database (CNKI) and WanFang Database from database inception to February 2023 using the terms of “mindfulness”, “burnout” and “medical students”. Two reviewers independently reviewed the studies, and extracted the data of the eligible studies, as well as assessed the risk of bias. A random-effects model was employed to calculate the standardized mean differences (SMD) with 95% confidence intervals (CI) of overall burnout and its sub-domains of burnout (i.e., emotional exhaustion, cynicism, and academic efficacy).

**Results:**

Of 316 records in total, nine studies (with 810 medical students) were ultimately included. The four RCT studies demonstrated an overall judgment of some concerns risk of bias, and the overall risk of biases of the five qRCT studies were judged as serious. In term of the SORT, the RCT and qRCT studies were evaluated as level 2 evidence, and the overall strength of recommendation was classified as B (limited-quality patient-oriented evidence). The pooled analysis showed that MBIs were associated with significant small to moderate improvements for medical students’ overall burnout (SMD=-0.64; 95% CI [-1.12, -0.16]; *P* = 0.009) in the included four RCTs, emotional exhaustion (SMD=-0.27; 95% CI [-0.50, -0.03]; *P* = 0.03) and academic efficacy (SMD = 0.43; 95% CI [0.20, 0.66]; *P*<0.001) in the four qRCTs.

**Conclusions:**

MBIs can serve as an effective approach for reducing burnout symptoms in medical students. Future high-quality studies with a larger sample size and robust randomized controlled trial methodologies should be obtained to reinforce the effectiveness of MBIs for reducing academic burnout in medical students.

## Background

A fast-growing body of studies is providing that burnout can be seen in medical students prevalently and then progressively develops over the course of medical education [1–3]. Burnout is a common term that was initially introduced among human service employees and subsequently investigated among other professionals and students [4]. Recently, researchers have not only sought to investigate burnout in a wide range of occupations, but also paid great attention on student population. As is known, medical students receive much longer and more arduous educational training compared with general college students. Medicine is a profession related to human life, which does not tolerate errors. According to a report in a systematic review, the prevalence of burnout among medical students ranged from 33.4 to 55.0% [5]. During the preclinical training, medical students are exposed to numerous academic and psychosocial stressors, e.g., heavy workload, high-demand academic achievements, and peer competition, leading to higher likelihood to suffer from burnout [6, 7]. Burnout may result in many adverse consequences if not appropriately addressed, which includes but not limited to depression, suicidal ideation, insomnia, thoughts of dropping out of medical school, increased drug or alcohol dependence/abuse [8–10]. What is worse, burnout may predispose medical students to an unprofessional situation, which can potentially place patients in peril [11].

Burnout is defined as a syndrome of emotional exhaustion, depersonalization and low personal accomplishment [12]. Maslach Burnout Instrument (MBI) was developed and employed to examine burnout in the three subscales including exhaustion, depersonalization and professional efficacy [5]. When the targeted population changed to students, another term “academic burnout” was adopted to highlight the core ingredients of burnout including emotional exhaustion, cynicism, and academic inefficacy [13]. Therefore, the MBI-Student Survey (MBI-SS) was developed to better precisely measure burnout among students, which is compatible to the specific circumstances encountered by students in an academic context [13]. In the MBI-SS, the subscale for emotional exhaustion examines fatigue caused by studies, the subscale for cynicism evaluates the indifference in student attitudes toward their studies, and the subscale for academic efficacy focuses on the academic accomplishment. The MBI-SS has proven to have good reliability and validity to measure student burnout across a number of countries [14–16].

Mindfulness is described as a process of paying attention to the present moment purposefully and nonjudgmentally [17], which can be trained through mindfulness-based interventions (MBIs). The parameter of MBIs varies in different studies they refer to, such as length, frequency, and delivery format. In recent years, the popularity of MBIs has grown rapidly due to the increasing evidence showing their effectiveness for diverse psychological and physical disorders, including burnout, depression, anxiety, stress, and chronic pain across a wide range of populations [18–20]. Although there was a large quantity of systematic review and meta-analysis reporting the effectiveness of MBIs, the population they focused on was primarily the healthcare professionals (e.g., physicians and nurses) and the outcomes broadly reflected the psychological syndromes (e.g., depression, anxiety and stress) rather than the specific aspects of burnout. Nevertheless, to the best of our knowledge, there is no prior systematic review and meta-analysis examining the pooled effect of MBIs on the overall burnout and their three compartments (i.e., emotional exhaustion, cynicism, and academic efficacy) in medical students. So, synthesizing and analyzing the current studies to secure a more precise estimate of the effects of MBIs on burnout is crucial for providing the evidence of implementing MBIs in medical students. Therefore, the systematic review and meta-analysis aimed to synthesize the scientific evidence and quantify the pooled effect of MBIs on the burnout syndrome in medical students.

## Methods

### Search strategy

This systematic review was strictly performed in accordance with the Preferred Reporting Items for Systematic Reviews and Meta-Analyses (PRISMA) guidelines [21]. The protocol for this systematic review can be found on PROSPERO (ref: CRD42023388097, available from https://www.crd.york.ac.uk/prospero/display_record.php?ID=CRD42023388097). A comprehensive literature search was conducted in the databases, including PubMed, Embase, ERIC, PsycINFO, Scopus, Cochrane Central Register of Controlled Trials (CENTRAL), China National knowledge Information Database (CNKI) and WanFang Database from database inception to February 2023 without any language restrictions. The main search terms were “mindfulness”, “burnout”, “medical students” in various combinations. For example, the search strategy in PubMed database was as follows: (((“Burnout, Psychological“[Mesh] OR “Burnout, Professional“[Mesh]) OR (((Burnout[Title/Abstract]) OR (Burnout Syndrome[Title/Abstract])) OR (Academic Burnout[Title/Abstract]))) AND ((“Mindfulness“[Mesh]) OR (Mindfulness*[Title/Abstract]))) AND ((“Students, Medical“[Mesh]) OR (((Medical Students[Title/Abstract]) OR (Student, Medical[Title/Abstract])) OR (Medical Student[Title/Abstract]))). The references from the already-retrieved literature were also searched to find additional articles of interest.

### Eligibility criteria

The PICO tool, endorsed by the Cochrane Collaboration, is the acronym of the Population, Intervention, Comparison and Outcomes of an article, which is universally employed to identify compartments of clinical evidence for systematic reviews and meta-analysis. However, the PICO tool has been modified to “PICOS” where the “S” implies the Study design due to the fact that qualitative research or qualitative designs were not specifically identified [22]. The inclusion criteria were set according to the PICOS guidelines: (1) Participants (P): participants were medical students regardless of their specialty, such as psychology, nursing, surgery, etc.; (2) Intervention (I): any type of mindfulness-focused interventions was included, such as breathing, body scanning, contemplation meditation exercises, yoga, etc.; (3) Comparison (C): any type of comparison conditions was considered, such as active intervention, nonactive intervention, waitlist; (4) Outcome: three sub-indicators of burnout syndrome involved emotional exhaustion, depersonalization and personal accomplishment measured by the Maslach Burnout Inventory and the Oldenburg Burnout Inventory; (5) Study design (S): quantitative research design including randomized controlled trials (RCTs) and non-RCTs.

### Study selection and data extraction

The research results were exported to Endnote 20.0 and the duplicates were identified and removed automatically. Two reviewers (Y.T.H. and J.G.) independently screened all titles and abstracts. The full-text review was subsequently conducted by the same two independent reviewers. Disagreements were resolved through in-depth review and discussion, and a third reviewer (Z.Z.W) was invited to reach a consensus if necessary. The extracted information of each study included: (1) characteristics of the study (i.e., authors, country, year of publication, study design); (2) characteristics of the population (i.e., sample size, sex, age, race/ethnicity, studying university); (3) characteristics of the intervention (i.e., treatment setting, treatment length, treatment frequency and type of delivery); (4) characteristics of the outcomes (i.e., means and standard deviations of three sub-indicators of burnout syndrome). If the extracted data were not reported in the article, we emailed the first/corresponding author/authors of that article to secure the missing data.

### Quality assessment and strength of recommendation

The quality of the eligible studies was assessed by two reviewers (Z.Z.W. and P.Y.W) independently, and the disagreements were resolved by thorough review and discussion until the consensus was reached. For RCTs, we used the revised Cochrane risk-of-bias tool for randomized trials (RoB 2.0) [23], which comprises five domains: (1) the randomization process, (2) deviations form intended interventions, (3) missing outcome data, (4) measurement of the outcome, and (5) selection of the reported results. The risk of bias of each included study is categorized as “low risk”, “some concerns” or “high risk”. Furthermore, the risk of bias of non-RCTs was evaluated by the Risk of Bias in Non-Randomized Studies of Interventions (ROBINS-I) across seven domains [24]: (1) confounding, (2) selection of participants, (3) classification of interventions, (4) deviations from intended interventions, (5) missing data, (6) measurement of outcomes, and (7) selection of the reported results. The risk of bias in each domain is rated as “low risk”, “moderate risk”, “serious risk”, “critical risk”, and “no information”. In term of the level of evidence and strength of recommendation, we used the Strength of Recommendation Taxonomy (SORT) [25], which classifies each study into three levels from 1 to 3 (level 1: good-quality patient-oriented evidence; level 2: limited-quality patient-oriented evidence; level 3: other evidence) and three strengths of recommendation from A to C (A: recommendation based on consistent and good-quality patient-oriented evidence; B: recommendation based on inconsistent or limited-quality patient-oriented evidence; C: recommendation based on consensus, usual practice, opinion, disease-oriented evidence, or case series for studies of diagnosis, treatment, prevention, or screening).

### Statistical analysis

The Review Manager software (RevMan v5.4, Cochrane Collaboration, Oxford, UK) was employed to conduct the data analysis. A random-effects model (DerSimonian–Laird approach) was used to determine the effects of mindfulness-based interventions on alleviating academic burnout of medical students by computing the standardized mean differences (SMD) with 95% confidence intervals (CI) [26]. We used the inverse variance method to weigh the studies. The value of the SMD was rated as small (from 0.2 to 0.49), moderate (from 0.50 to 0.79), or large (equal to or greater than 0.80) [27]. Besides, we used inconsistency test (I²) to examine the heterogeneity between included studies [28]. It is commonly accepted that I² values above 25%, 50%, and 75% were interpreted as low, moderate, and high heterogeneity, respectively. *P* < 0.05 was considered as statistical significance.

## Results

### Study search results

**Fig. 1:**
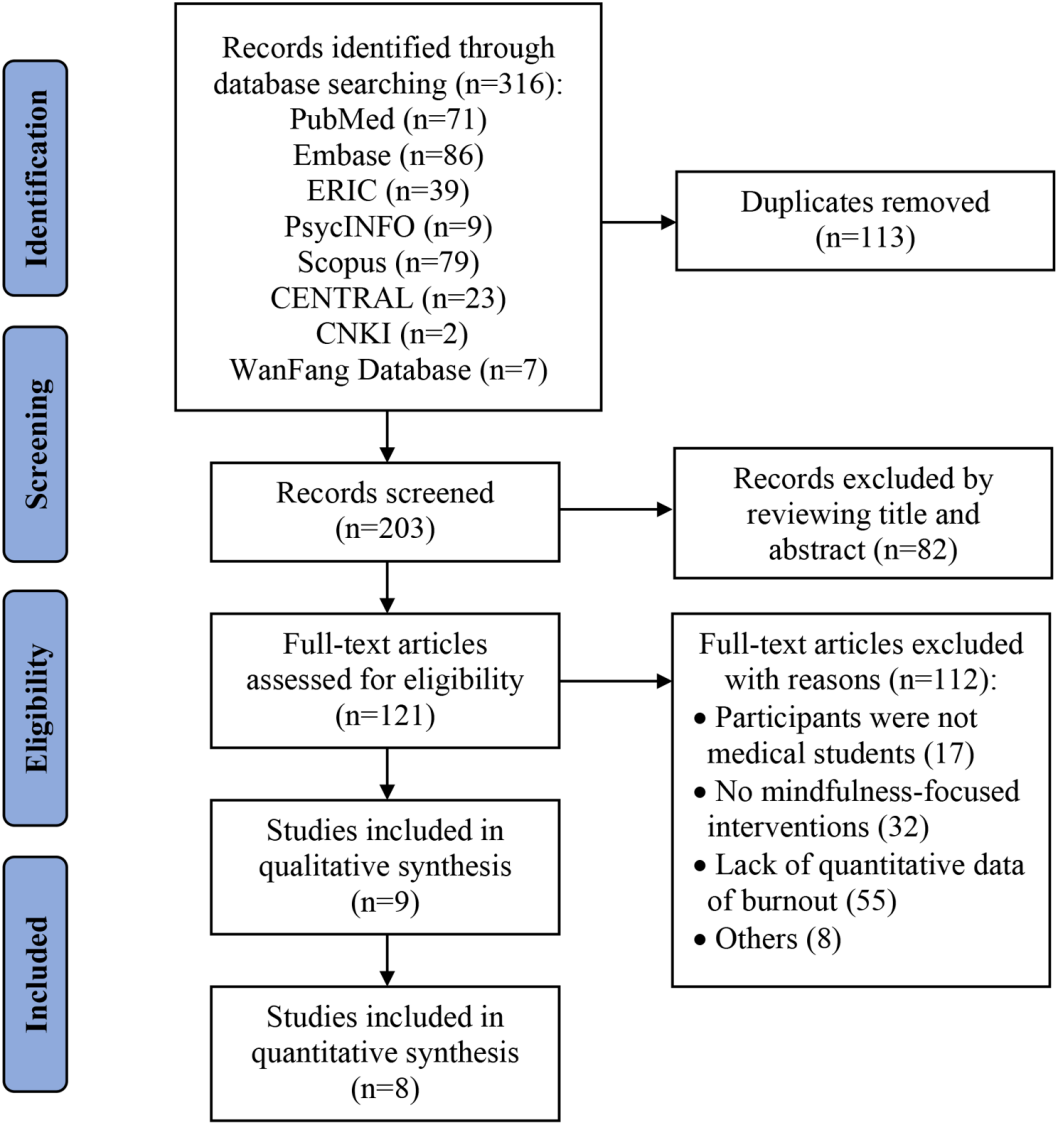
PRISMA flowchart for study selection

Our searches yielded 316 records in total (seen in Fig. 1). After removing duplicates, we screened the titles and abstracts of the remaining 203 records, of which 121 were determined to be eligible for full-text screening. A total of nine articles met the inclusion criteria for the qualitative analysis [29–37]. For the meta-analysis, one study was excluded due to the inaccessibility of extracting the data [37].

### Characteristics of the included studies

The characteristics of the included studies were demonstrated in Table 1. The year of publication of included studies ranged from 2013 to 2021. Three studies were conducted in China [29–31], two in Ireland [33, 34], one in Norway [32], one in Spain [35], one in the United Kingdom [36], one in the United State [37]. All studies were reported in English, except for 3 studies in Chinese [29–31]. A total of 810 participants were included in the qualitative review. Four RCTs and five qRCTs analyzed the effect of MBIs on burnout in medical students. The included studies employed a variety of intervention categories. Two studies used the Mindfulness-Based Stress Reduction (MBSR) course [32, 37], five studies used the modified MBSR course [29–31, 34, 35], one study used online version of MBSR programme [33], and one study used “Five-week Living Mindfully MBSR” [36]. The length of the MBIs ranged from 2 to 16 weeks. The MBIs involved diverse mindfulness-based practices, such as breathing, body scanning, contemplation meditation exercises, didactic exercises, yoga, and dialogue groups. In term of the outcome measures, three studies used the 20-item Learning Burnout of Undergraduate Students (LBUS) [29–31], three studies used the 15-item Maslach Burnout Inventory-Student Survey (MBI-SS) [32, 35, 36], three studies used the 16-item Maslach Burnout Inventory (MBI) [33, 34, 37]. Four studies reported the statistically significant difference of burnout after receiving MBIs [29–31, 33].

**Table 1:** Characteristics of the included studies

Study, year, country	Design	Participants	Mindfulness-based interventions	Burnout measures	Results
Ye et al. [29], China	RCT	60 medical students (30 in the intervention and control group respectively), age range was 19 ∼ 22 years, 61.7% participants were female	Modified MBSR course of 2 weeks with 14 sessions of 0.5-hour duration each, including breathing, mindfulness practice, dialogue groups	Learning Burnout of Undergraduate Students (LBUS) including 20 items evaluated on a 5-point Likert scale (1 ∼ 5)	Significant effect was observed of MBIs on burnout score
Zhao et al. [30], China	RCT	60 medical students (30 in the intervention and control group respectively), mean age was 19.32 (SD = 1.65) and 19.16 (SD = 1.38) in the intervention and control group respectively, 48.3% participants were female	Modified MBSR course of 8 weeks with 8 sessions of 1.5-hour duration each, including breathing, mindfulness practice, yoga, dialogue groups	Learning Burnout of Undergraduate Students (LBUS) including 20 items evaluated on a 5-point Likert scale (1 ∼ 5)	Significant effect was observed of MBIs on burnout score
Sun [31], China	RCT	63 medical students (31 in the intervention group and 32 in the control group), age range was 15 ∼ 17 years, 53.97% participants were female	Modified MBSR course of 6 weeks with 6 sessions of 1.5-hour duration each, including breathing, mindfulness practice, yoga, dialogue groups	Learning Burnout of Undergraduate Students (LBUS) including 20 items evaluated on a 5-point Likert scale (1 ∼ 5)	Significant effect was observed of MBIs on burnout score
de Vibe et al. [32], Norway	RCT	288 medical students (144 in the intervention and control group each), mean age was 23 years, 76% participants were female	The MBSR programme of 8 weekly sessions with 2.5 h each, including physical and mental exercises, didactic teaching and group process	Maslach Burnout Inventory-Student Survey (MBI-SS) including 15 items evaluated on a 7-point Likert scale (0–6)	No statistically significant difference was observed
O’Driscoll, Byrne et al. [33], Ireland	qRCT	52 undergraduate pharmacy students (19 in the intervention group and 33 in the control group), age range was 18 ∼ 24 years, 76.9% participants were female	An online introductory version of the face-to-face MBSR course of 4 weekly sessions with one-hour online class and 20-minute daily practice each, including body scans, awareness of breathing, mindful enquires, and group activities	Maslach Burnout Inventory Student Survey (MBI-SS) University Form including 16 items evaluated on a 7-point Likert scale (0–6)	Significant effect was observed of MBIs on the academic efficacy only
O’Driscoll, Sahm et al. [34], Ireland	qRCT	99 undergraduate pharmacy students (51 in the intervention group and 48 in the control group), age range was 18 ∼ 24 years, 66.7% participants were female	Modified MBSR course of 6 weeks with 6 sessions of 2-hour duration and 20-minute daily practice each, including breathing, mindful enquires, and group activities	Maslach Burnout Inventory Student Survey (MBI-SS) University Form including 16 items evaluated on a 7-point Likert scale (0–6)	No effect was observed of MBIs on burnout score
Oró et al. [35], Spain	qRCT	143 medical students (68 in the intervention group and 75 in the control group), Mean age was 20.28 (SD = 1.54) years, 73.4% participants were female, 20.3% participants had prior mindfulness-related knowledge	Modified MBSR course of 16 weeks with 8 sessions of 2-hour duration each, including contemplation, meditation, exercises, mindfulness practice and yoga	Maslach Burnout Inventory-Student Survey (MBI-SS) including 15 items evaluated on a 7-point Likert scale (0–6)	No effect was observed of MBIs on burnout score
Clarkson et al. [36], UK	qRCT	14 medical students (8 in the intervention group and 6 in the control group), mean age was 30 and 26 in the intervention and control group respectively, 42.9% participants were female	Five-week Living Mindfully MBSR programme which is based on the original Kabat-Zin course	Maslach Burnout Inventory Student Survey (MBI-SS) including 15 items evaluated on a 7-point Likert scale (0–6)	No statistically significant difference was observed, but almost 1/3 of the participants experienced burnout
Barbosa et al. [37], US	qRCT	31 medical students (16 in the intervention group and 15 in the control group),	The MBSR programme of 8 weekly sessions with 2.5 h each, including formal mindfulness practice, formal meditation practice, informal practices, group process	Maslach Burnout Inventory (MBI) including 16 items evaluated on a 7-point Likert scale (0 ∼ 6)	No statistically significant difference was observed

MBSR: Mindfulness-Based Stress Reduction; RCT: Randomized Controlled Trial; qRCT: quasi-randomized controlled trial

### Risk of bias, level of evidence, and strength of recommendation

As shown in Table 2, Four RCT studies [29–32] demonstrated an overall judgment of some concerns risk of bias. Specifically, the overall some concerns risk of bias was found to be from the bias in the deviations from intended intervention and the measurement of outcome. In term of the SORT, the RCT studies were evaluated as level 2 evidence, and the overall strength of recommendation was classified as B (limited-quality patient-oriented evidence). Regarding the qRCTs [33–36], the overall risk of bias was judged as serious, which mainly derived from the bias in the measurement of the outcome according to ROBINS-I (presented in Table 3). In term of the SORT, the qRCT studies were rated as level 2 evidence, and the overall strength of recommendation was classified as B (limited-quality patient-oriented evidence).

**Table 2:** Risk of bias, level of evidence, and strength of recommendation for RCTs

Study, year	Randomization process	Deviations from intended intervention	Missing data	Measurement of outcome	Selection of reported results	Overall risk of bias	Level of evidence	Strength of recommendation
Ye et al., [29]	Low risk	Some concerns	Low risk	Some concerns	Low risk	Some concerns	2	B
Zhao et al., [30]	Low risk	Some concerns	Low risk	Some concerns	Low risk	Some concerns	2	B
Sun, [31]	Low risk	Some concerns	Low risk	Some concerns	Low risk	Some concerns	2	B
de Vibe et al., [32]	Low risk	Some concerns	Low risk	Some concerns	Low risk	Some concerns	2	B

**Table 3:** Risk of bias, level of evidence, and strength of recommendation for qRCTs

Study, Year	Confounding	Selection classification of participants	Classification of intervention	Deviations from intended intervention	Missing data	Measurement of outcome	Selection of reported result	Overall risk of bias	Level of evidence	Strength of recommendation
O’Driscoll, Byrne et al., [33]	Moderate risk of bias	Moderate risk of bias	Low risk of bias	Moderate risk of bias	Low risk	Serious risk of bias	Low risk	Serious risk of bias	2	B
O’Driscoll, Sahm et al., [34]	Moderate risk of bias	Moderate risk of bias	Low risk of bias	Moderate risk of bias	Low risk	Serious risk of bias	Low risk	Serious risk of bias	2	B
Oró et al., [35]	Moderate risk of bias	Moderate risk of bias	Low risk of bias	Moderate risk of bias	Low risk	Serious risk of bias	Low risk	Serious risk of bias	2	B
Clarkson et al., [36]	Moderate risk of bias	Moderate risk of bias	Low risk of bias	Moderate risk of bias	Low risk	Serious risk of bias	Low risk	Serious risk of bias	2	B

### Meta-analysis results

**Fig. 2:**
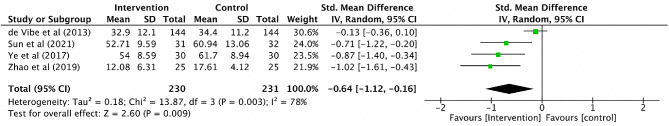
Forest plot for the overall burnout

**Fig. 3:**
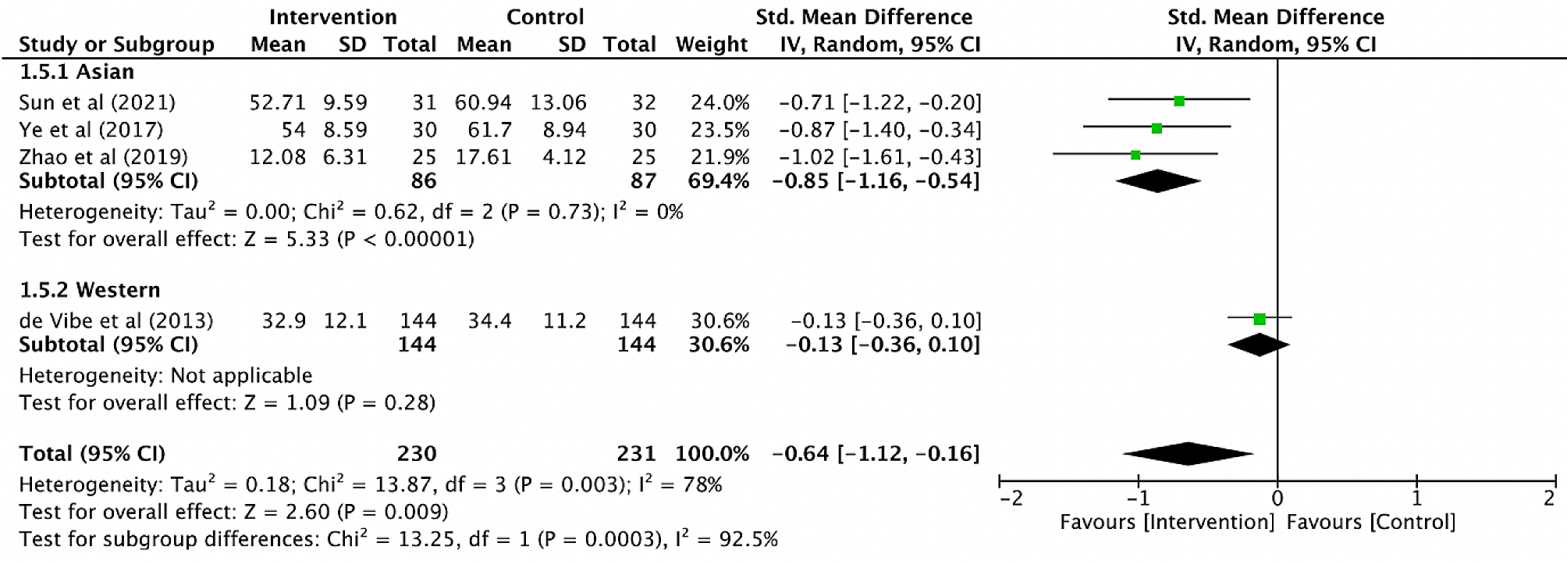
Forest plot for the sub-group analysis of the overall burnout

As shown in Fig. 2, four RCTs [29–32] analyzed the total score of burnout, including a total of 461 participants (230 in the MBIs group and 231 in the control group). The pooled analysis revealed a statistically significant moderate effect of MBIs on the overall burnout (SMD=-0.64; 95% CI [-1.12, -0.16]; *P* = 0.009). However, due to the relatively high heterogeneity (I^2^ = 78%), a sensitivity analysis was conducted where the included studies were removed separately. The heterogeneity was significantly reduced when the study of de Vibe et al. (2013) was excluded (SMD=-0.85; 95% CI [-1.16, -0.54]; *P*<0.001; I^2^ = 0%). On the subgroup meta-analysis, there was a significant difference between Asian sub-group and Western sub-group (*P*<0.001) (Fig. 3).

**Fig. 4:**
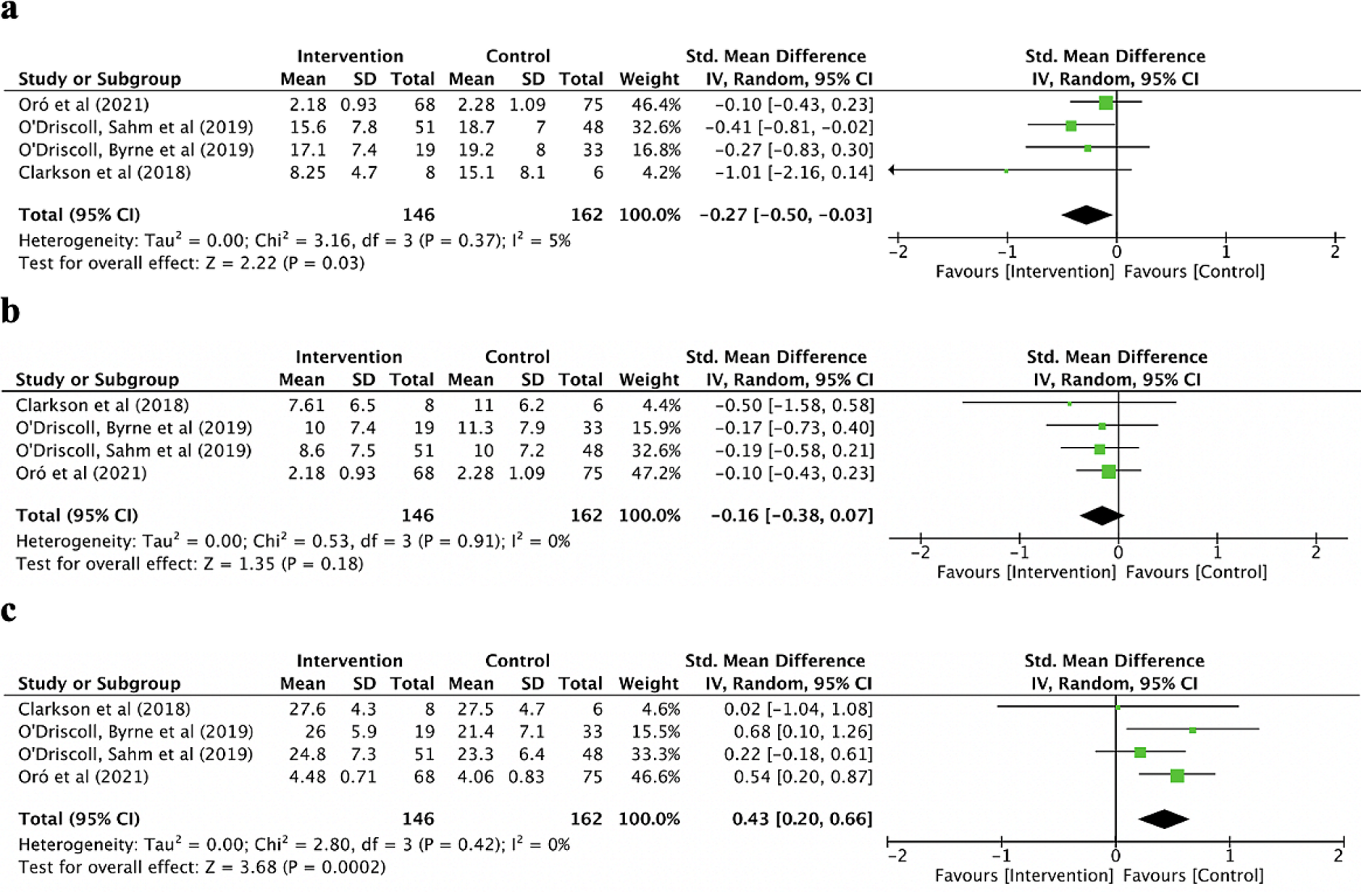
Forest plot for the sub-domains of burnout. **a** the emotional exhaustion of burnout. **b** the cynicism of burnout. **c** the academic efficacy of burnout

As presented in Fig. 4, four qRCTs [33–36] analyzed the emotional exhaustion, cynicism and academic efficacy of burnout, including a total of 308 participant (146 in the MBIs group and 162 in the control group). The pooled result revealed a significant small effect of MBIs on emotional exhaustion (SMD=-0.27; 95% CI [-0.50, -0.03]; *P* = 0.03; I^2^ = 5%) and academic efficacy (SMD = 0.43; 95% CI [0.20, 0.66]; *P*<0.001; I^2^ = 0%) (Fig. 4a & c). However, the pooled analysis revealed that the improvement of cynicism in the MBIs group was not significantly different from the control group (SMD=-0.16; 95% CI [-0.38, 0.07]; *P* = 0.18; I^2^ = 0%) (Fig. 4b).

## Discussion

The research of MBIs in medical students has skyrocketed in recent years. To be best of our knowledge, this is the first systematic review and meta-analysis of current studies evaluating the effectiveness of MBIs to alleviate burnout in medical students. A total of 8 studies included in the meta-analysis, suggesting that MBIs had significant small to moderate effects on reducing overall burnout and its two sub-domains (i.e., the emotional exhaustion and academic efficacy). However, the effects of MBIs on the cynicism of burnout were not statistically significant. Although previous reviews and meta-analysis also reported the beneficial effects of MBIs in reducing burnout [38–40], the population they targeted was healthcare professionals rather than medical students. The study of Daya and Hearn (2018) synthesized the impact of MBIs on medical students for psychological symptoms [41]. For the burnout aspect, only three studies were included in this review, which concluded that no significant reductions were observed due to lack of sufficient high-quality studies included. Nevertheless, despite a total of 8 studies included in the meta-analysis, we need to be cautious of the results due to the overall high risk of bias and limited-quality evidence observed in the included studies. In the meanwhile, it is noted that the contents and durations of MBIs were not consistent across studies, and the effects also varied. For example, Zhao et al., found that there was a significant difference between medical students receiving or not receiving the modified MBSR course of 8 weeks with 8 sessions of 1.5-hour duration each, including breathing, mindfulness practice, yoga, dialogue groups [30]. While de Vibe et al. failed finding the significant results for medical students who received the MBSR programme of 8 weekly sessions with 2.5 h each, including physical and mental exercises, didactic teaching and group process [32].

The results showed that MBIs can reduce the emotional exhaustion of medical students in the included four qRCT studies. A large amount of literature supported that emotional exhaustion can be considered as one of the core elements of burnout [4, 42, 43]. According to a national survey, medical students were more likely to experience high emotional exhaustion and low academic efficacy than early career physicians (44.6% versus 39.6%) [44]. Likewise, a meta-analysis reported a prevalence of 40.8% (95%CI: 32.8%∼48.9%) of high emotional exhaustion in the included 7588 medical students [5]. Given that high emotional exhaustion is correlated with high psychiatric morbidity [45], medical students should be informed about the importance of preventing/alleviating emotional exhaustion through evidence-based interventions, such as MBIs. Previous meta-analysis only reported the effectiveness of MBIs on the overall burnout score and found no substantial difference post-intervention for burnout (SMD=-0.42; 95% CI [-0.84, 0.00]; *p* = 0.05; I^2^ = 0%) [46]. Our results provided the evidence that MBIs can significantly reduce the overall burnout and emotional exhaustion in medical students. Therefore, it is MBIs that served as an effective approach to prevent or reduce overall burnout and emotional exhaustion in medical students.

The meta-analysis revealed a non-significant small reduction in the cynicism domain of burnout for medical students receiving MBIs in the included qRCT studies. Cynicism is regarded as a medical student’s detached feelings and impersonal treatment or negative attitudes towards others [47]. It was reported that the prevalence of cynicism reached 35.1% (95%CI: 27.2%∼43.0%) among 7588 medical students [5]. Considering the positive association between cynicism and psychiatric morbidity [45], medical organizations or institutes should alert medical students of the importance of preventing/alleviating cynicism through evidence-based approaches, such as MBIs. The estimate of effect supported the MBIs in reducing cynicism in the included four qRCTs, albeit the non-significant difference between the MBIs group and the control group. Thus, future more studies are needed to strengthen the evidence of MBIs for improving the cynicism domain of burnout in medical students.

For the academic efficacy domain of burnout, it is suggested that MBIs can significantly enhance academic efficacy of medical students in the included four qRCT studies. Academic efficacy refers to the feeling of being effective in the study and being promoted for the position [11]. As reported in a meta-analysis, the prevalence of academic efficacy got to 27.4% (95%CI: 20.5%∼34.3%) in a number of 7588 medical students [5]. Compared with the other domains of burnout, the prevalence of academic inefficacy is relatively low. But in practice, burnout is seen as an intertwined unity rather than three separate compartments. So, addressing the academic inefficacy domain of burnout is also of importance. Our findings supported the effectiveness of MBIs in alleviating the academic inefficacy domain of burnout.

The strength of this study is that, to our best knowledge, it is the first systematic review and meta-analysis to determine the pooled effect of MBIs for alleviating overall burnout and its sub-domains in medical students. The findings of this study can provide evidence of MBIs for burnout prevention/reduction among medical students in academic milieu. However, there are some limitations we need to address in this study. First of all, a small number of 8 studies included in the meta-analysis, of which only 4 studies were RCT design. Hence, the results should be interpreted cautiously. Secondly, the publication bias was not evaluated due to fewer than ten studies included in the meta-analysis. Thirdly, for the subgroup meta-analysis of the overall burnout, we divided articles into Asian and Western groups based on the source of population. However, the Asian group merely included articles found in Chinese database, and we did not search a database in the language of another Western country specifically due to the language barrier. Thus, the results of this sub-group meta-analysis may have some biases. Lastly, the risk of bias of the included studies was relatively high, and the overall evidence and strength of recommendation were rated as limited-quality patient-oriented evidence. Therefore, more high-quality studies with robust randomized controlled trial methodologies are needed to minimize the risk of bias existing in the included studies and enhance the strength of recommendation of MBIs for reducing burnout in medical students.

## Conclusion

To sum up, the results of this systematic review and meta-analysis suggested that MBIs can serve as an effective approach for reducing burnout symptoms in medical students. Future high-quality studies with a larger sample size and robust randomized controlled trial methodologies should be obtained to reinforce the effectiveness of MBIs for reducing academic burnout in medical students.

## Data Availability

The datasets used and/or analyzed during the current study available from the corresponding author/first author on reasonable request.

## Abbreviations

MBIs: Mindfulness-Based Interventions
MBSR: Mindfulness-Based Stress Reduction
MBI: Maslach Burnout Instrument
MBI-SS: Maslach Burnout Instrument-Student Survey
LBUS: Learning Burnout of Undergraduate Students
RCT: Randomized Controlled Trial
RoB 2.0: Revised Cochrane Risk-of-Bias Tool for Randomized Trials
SORT: Strength of Recommendation Taxonomy
ROBINS-I: Risk of Bias in Non-Randomized Studies of Interventions
SD: Standard Deviation
SMD: Standardized Mean Differences
CI: Confidence Intervals
